# A Cross-Sectional Study Examining Youth Smoking Rates and Correlates in Tbilisi, Georgia

**DOI:** 10.1155/2014/476438

**Published:** 2014-03-13

**Authors:** Carla J. Berg, Ana Aslanikashvili, Mamuka Djibuti

**Affiliations:** ^1^Department of Behavioral Sciences & Health Education, Emory University Rollins School of Public Health, 1518 Clifton Road NE, Atlanta, GA 30322, USA; ^2^International School of Public Health, Tbilisi State Medical University, 7 Mikheil Asatiani Street, Tbilisi, Georgia; ^3^RTI International, Georgia HIV Prevention Project, 7 Mikheil Asatiani Street, Tbilisi, Georgia

## Abstract

Georgia has high smoking rates; however, little is known about the prevalence and correlates of youth smoking. We conducted a secondary data analysis of a 2010 cross-sectional survey of 1,879 secondary and postsecondary school students aged 15 to 24 years in Tbilisi, Georgia, examining substance use, perceived risk, and recreational activities in relation to lifetime and current (past 30 days) smoking. Lifetime and current smoking prevalence was 46.1% and 22.6%, respectively. In secondary schools, lifetime smoking correlates included being male, consuming alcohol, lifetime marijuana use, and lower perceived risk (*P*'s ≤ .001). Correlates of current smoking among lifetime smokers included being male, consuming alcohol, lifetime marijuana use, lower perceived risk, less frequently exercise, and more often going out (*P*'s < .05). In postsecondary schools, lifetime smoking correlates included being male, consuming alcohol, lifetime marijuana use, lower perceived risk, more often going out, and recreational internet use (*P*'s < .0). Correlates of current smoking among lifetime smokers included being male (*P*'s = .04), consuming alcohol, marijuana use, lower perceived risk, and more often going out (*P*'s < .05). Tobacco control interventions might target these correlates to reduce smoking prevalence in Georgian youth.

## 1. Introduction

There are an estimated 1.3 billion adult smokers among the world's six billion people, with increases anticipated [1]. Cigarette smoking is the second leading risk factor for death worldwide [2–4]. More than six million people die every year as a consequence of tobacco smoking [5]. In 2000, an estimated 4.83 million deaths were attributed to cigarette smoking globally. Tragically, almost half of those deaths occur in the developing world [2, 3]. In fact, four-fifths of current smokers live in low- and middle-income counties (LMICs) [5]. Many LMICs are still in early stages of the tobacco epidemic; thus, the number of smoking-related deaths in these nations is likely to increase [3, 6, 7]. Based on current trends, mortality will increase to 8.3 million a year by 2030, and 80% of these deaths will occur in LMICs [5].

One high-risk region for tobacco use is the area of the former Soviet Union [8]. In a study of eight former Soviet Union countries, almost 80% of men reported a history of smoking [8]. Rates of current smoking ranged from 43.3% in Moldova to 65.3% in Kazakhstan. There are drastically different smoking rates among men and women in these regions, with men having a much higher prevalence of smoking. In general, men from rural areas, of lower education and income, had higher rates of smoking, while women in urban areas and of higher education and income had higher smoking prevalence rates [8, 9].

The Republic of Georgia, one former Soviet Union country and a lower middle-income country [10, 11], has shown a record decrease in population over recent years, mainly attributed to premature mortality and migration [12]. The tobacco-related death toll in Georgia is estimated to be around 11,000 deaths per year [12]. Among Georgian men, estimated 54.9% are current daily smokers, 17.0% are less than daily smokers, and 28.1% are nonsmokers (past and never smokers) [12]. Among Georgian women, an estimated 12.2% are current daily smokers, 6.4% are less than daily smokers, and 81.4% are nonsmokers [12]. Similar to the trends of the other former Soviet Union countries, smoking prevalence is higher among men with lower education and lower income and among those who live in smaller settlements [12], whereas the smoking prevalence among women is higher among the more educated and affluent and those who live in larger cities. This may be a sign of the growing tobacco epidemic among Georgian women, such that rates of smoking among women grew from roughly 24% in 1997 to 34% in 2007 in Tbilisi, with the greatest increases among those under 40 years of age [12]. Given the growing tobacco use epidemic in Georgia, strides are being made to curtail this epidemic. In December 2005, Georgia ratified the Framework Convention on Tobacco Control (FCTC), which mandates that nations that ratify the FCTC implement policies including smoke-free public policies and regulation of tobacco advertising, among other tobacco control policies.

The Socioecological Model (SEM) [13–15] is a framework to examine the multiple effects and interrelatedness of environmental, contextual, and social factors on individual behavior. The SEM involves a comprehensive approach that integrates multiple levels of influence that impact health behavior and ultimately health outcomes. Those levels of influence include intra- and interpersonal factors, community and organizational factors, and public policies [13–15]. For example, some variables that might influence smoking may be intrapersonal factors such as sociodemographics, substance use behaviors, and attitudes toward smoking and smoking-related policies; interpersonal factors such as social exposure to smokers; community or organizational factors such as prevalence of smoking in their community, social norms within their community, or exposure to tobacco advertising; or public policies including those that regulate advertising, taxation, or smoke-free policies in public places. Drawing from this perspective, the current study focuses on individual-level factors including nonmodifiable factors such as sociodemographic characteristics as well as modifiable factors including substance use behaviors and involvement in activities with the potential for social influence on smoking initiation and maintenance among youth in Tbilisi, Georgia. These factors are impacted by the cultural context and social norms related to tobacco use in this understudied, high-risk country.

In terms of substance use behaviors, a large amount of literature has documented the association between cigarette use and alcohol use [16–18], as well as smoking and marijuana use [19, 20]. However, little research has documented these findings among youth in LMICs or specifically in Georgian youth. In particular, alcohol is a major concern among Georgian youth. One 2009 survey [21] found that more than 90% of youth have had drunk alcohol at least once, and more than 43% have had their last drink at home. This suggests a cultural acceptance of alcohol use in the Georgian society and within Georgian families. Given the high prevalence of both tobacco use and alcohol use in this context, the comorbid nature of behaviors perceived to be high risk in other cultures may not be associated among youth in Georgia.

Additionally, activity involvement may indicate specific risk or protective influences related to smoking behavior. For example, social factors have been found to play a particularly important role in smoking initiation and maintenance in other countries. Family influences, such as parents' smoking [22–25], and peer and school influences, such as peers' smoking [24, 26–28], are well- documented predictors of youth smoking. On the other hand, being married and having a family have been associated with lower likelihood of smoking in young adulthood in developed countries [29, 30]. In addition, low academic achievement [26, 31] and low school commitment or attachment [31] has been found to be risk factors for smoking among youth in developed countries. Additionally, engagement in sports or physical activity has been documented protective factors against smoking among youth [32, 33] as well as predictive of tobacco use [34, 35]. Finally, Internet use among youth has been a factor more recently examined and may indicate risk for exposure to tobacco advertising, as the Internet provides tobacco companies with a highly active environment to advertise their products in increasingly regulated countries [36, 37].

Despite the literature regarding risk factors for smoking among youth, limited research has documented these associations in LMICs or in Georgia in particular. Given the limited tobacco control policy adoption and enforcement in these countries including Georgia, the social norms regarding smoking may be more conducive to smoking initiation or may have little differential impact given the pervasive nature of tobacco use in the community and social networks of youth. Moreover, the pervasiveness of tobacco use may also impact the perceived harm of cigarette smoking, such that youth will perceive the threat to be lower [16, 38]. Thus, examination of social factors impacting smoking among youth in LMICs is critical in informing approaches aimed at smoking prevention and cessation.

Given the aforementioned literature, the specific aims of this study are to examine sociodemographics, other substance use, perceived risk of smoking, and engagement in various social and academic activities in relation to lifetime use of cigarettes and current (past 30 day) cigarette use among lifetime cigarette users in a sample of 15–18-year-old secondary school students and 18–24-year-old postsecondary school students in Tbilisi, Georgia. We hypothesize higher lifetime and current cigarette smoking rates among the older age group, males, alcohol and marijuana users, those perceiving less harm related to cigarette smoking, and those who more frequently engage in activities where social influence might promote smoking.

## 2. Materials and Methods

### 2.1. Ethics Statement

The current study is a secondary data analysis of the 2010 USAID-funded Georgia HIV Prevention Project Behavioral Surveillance Survey among School and University Students in Tbilisi. The Georgia HIV Prevention Project is a five-year effort that began February 4, 2010, designed to improve and expand upon HIV prevention among the highest risk populations. This study was conducted among 15–18-year-old secondary school students and 18–24-year-old postsecondary school students in Tbilisi in order to fill the gap in current data about the knowledge, attitudes, and behaviors of youth in Tbilisi. This research was approved by the Institutional Review Boards of the Research Triangle Institute and the Maternal and Child Care Union in Georgia. All participants were informed of the nature of the study prior to their participation. For participants under the age of 18 years, both written parental consent and the student's written consent were obtained. For students 18-year old and older, written consent was obtained. In addition, the youth were informed that at any time during the interview they had the freedom to refuse to answer a question or to quit the interview. Both institutional review boards approved these procedures.

### 2.2. Participants and Procedures

The statistical population of this study is students 15 to 24 years of age attending public (state) or private secondary schools (9th to 12th grades); undergraduates in private or public universities; or students in vocational-technical training schools in Tbilisi in 2011. The total number of secondary school students in Tbilisi was 55,842; the total number of postsecondary students in Tbilisi universities and professional vocational-technical training schools was 73,652 (per the Ministry of Education and Science as of September 2010).

The sample size calculation was done using the methodology for descriptive studies for an estimated percentage in the target population with the event of interest (e.g., lifetime sexual activity, lifetime alcohol consumption, lifetime cigarette use) of 50% (confidence interval (CI) of 0.10; confidence level of 95%), indicating a minimum of 384 students in each of the four gender and age (15–18 year olds; 18–24 year olds) groups. Next, estimation of the sample size was done for a comparison of proportions of dichotomous variables for alpha error = 0.05 (two-sided test), power = 80%, and the expected smaller proportion = 0.5 (again maximizing the sample size) for the detection of difference = 0.10. By this methodology, it was determined that a minimum of 407 students should be selected per each group to reach an adequate statistical significance for mutual comparisons of age and gender groups. Therefore, this study attempted to enroll 2,000 students in total, 500 per age and gender group, considering a potential 80% response rate. The probability proportional to size sampling technique was used for the selection of institutions; that is, we aimed to obtain subgroups of participants relative in size to the size of the subpopulation in the general population. As a result, 24 secondary schools (16 state and 8 private) and 13 universities and/or vocational-technical training schools (7 state, 5 private universities, and one vocational-technical training school) were selected. Data regarding the specific school from which the participants were recruited were not recorded in order to protect the privacy of the schools and the confidentiality of the individual data collected, particularly given the sensitive nature of the information (e.g., drug use, sexual activity).

Refusal rates for school students and postsecondary students were 5.1% and 1.3%, respectively. Among the 1,936 completed questionnaires, 41 were excluded because of ineligible ages (less than 15 or more than 24 years of age), 3 were excluded because they were incomplete (almost half of the questions were left unanswered), and 13 were excluded due to doubtful responses (e.g., inconsistent or illogical responses), leaving a total of 1,879 useable questionnaires.

### 2.3. Measures

The survey assessed the following factors: sociodemographics, tobacco use, alcohol and marijuana use, knowledge of HIV/AIDS, sexual behavior, perceived risk of smoking, and activity involvement. For the current study, the following assessments were included.

#### 2.3.1. Sociodemographics

Participants were asked to indicate their age and gender. Employment and marital status were also assessed among the postsecondary school students.

#### 2.3.2. Cigarette Smoking

Participants were asked, “Have you ever smoked a cigarette?” and “How often have you smoked cigarettes over the last month? Have not smoked at all; less than 1 cigarette per week; less than 1 cigarette per day (cpd); 1–5 cpd; 6–10 cpd; 11–20 cpd; or more than a pack a day.” These measures were adapted from other international surveys including the European School Survey Project on Alcohol and Other Drugs [39] and the Global Youth Tobacco Survey [40].

#### 2.3.3. Alcohol Use

Participants were asked, “Have you ever had an alcoholic drink (wine, beer, vodka, martini, champaign, other drink containing alcohol)?” and “Have you had an alcoholic drink over the past month?” These questions are adapted from other validated surveys [39, 41, 42]. For the current analysis, we used the latter question to indicate alcohol use given the high prevalence of lifetime use (91.9%) versus past 30-day use of alcohol (64.8%).

#### 2.3.4. Marijuana Use

Participants were asked, “On how many occasions (if any) have you smoked marijuana or hashish - In your lifetime? In the past 12 months? In the past 30 days?” These assessments were adapted from other validated surveys [39, 41, 42]. Given the low prevalence of past 30 day use (1.0%) and past 12 month use (4.3%), lifetime marijuana use (11.1%) was included in the current analyses.

#### 2.3.5. Perceived Risk

Participants were asked, “In your opinion, how much do you think people risk harming themselves (physically, emotionally, or in other ways) if they…Smoke cigarettes sometimes? Smoke less than 10 cigarettes daily? Smoke around 10–20 cigarettes daily? Smoke a pack or more daily?” with response options of 1 =* no risk *to 4 =* great risk*. This assessment was adapted from the European School Survey Project on Alcohol and Other Drugs [39]. Given the high internal consistency of the items (Cronbach's alpha = 0.76), these four questions were used as a single measure of perceived risk.

#### 2.3.6. Activity Involvement

Participants were asked about the frequency with which they engaged in several activities. These six items asked, “How often have you done the following: Read fiction literature for entertainment? Engaged in sports or physical exercising? Went to parties, cafes, bars, or disco in the evening? Used the internet to listen to music, play, or chat? Used the internet for educational or work purposes? Went out in the neighborhood street and pass time with neighborhood friends or neighbors?” with response options of 1 =* never *to 5 =* almost every day*. These questions were adapted from the European School Survey Project on Alcohol and Other Drugs [39] and may be indicators of the degree to which social influence on smoking behaviors may be encountered.

### 2.4. Data Analysis

Participant characteristics were summarized using descriptive statistics. Bivariate analyses were conducted to identify correlates of lifetime cigarette use and, among lifetime smokers, current (past 30 day) cigarette smoking among 15–18-year-old secondary school students and among 18–24-year-old postsecondary school students, respectively. Chi-squared tests were used for categorical variables, and independent samples* t*-tests were used for continuous variables. Binary logistic regression was used to examine factors associated with lifetime cigarette use and, among lifetime users, current cigarette smoking among 15–18-year-old secondary school students and among 18–24-year-old postsecondary school students, respectively. Drawing from the literature, we forced sociodemographic characteristics and substance use into each of the multivariate regression models. Then, other factors including perceived harm and activity involvement that were associated with cigarette use at the *P* < .10 were entered using backwards stepwise entry. We also explored interaction effects, specifically gender by activity involvement. SPSS 21.0 was used for all data analyses. Statistical significance was set at *α* = .05 for all tests.

## 3. Results

In terms of cigarette use, slightly less than one-half (46.1%) had ever smoked (not shown in tables). Males were significantly more likely to have smoked than females (62.2% versus 33.0%; *P* < .001), and postsecondary students (50.6%) were more likely to have smoked than secondary school students (43.8%; *P* < .001). Of all participants, 22.6% reported smoking in the last month before the survey. One-third (33.9%) of males compared with 11.9% of females had smoked in the past 30 days (*P* < .001), and 28.3% of students of 18–24 years had smoked in the past 30 days compared with 17.6% of secondary school students (*P* < .001). Among past 30-day smokers, 15.5% smoked less than 1 cigarette per week, 6.6% smoked less than 1 cpd, 22.4% smoked 1–5 cpd, 20.0% smoked 6–10 cpd, 25.9% smoked 11–20 cpd, and 9.6% smoked more than a pack per day. Daily smoking in the last month was reported by 17.6% of all participants. Again, males (28.6%) were significantly more likely to have smoked daily in the last month than females (7.2%; *P* < .001), and postsecondary school students 18–24 years of age (23.7%) were significantly more likely to be daily smokers than secondary students 15–18 years of age (11.2%; *P* < .001). In terms of perceived risk, participants reported greater risk with greater consumption: smoking sometimes (*M* = 2.57, SD = 0.96), smoking less than 10 cpd (*M* = 3.06, SD = 0.81), smoking around 11–20 cpd (*M* = 3.61, SD = 0.69), and smoking a pack or more daily (*M* = 3.79, SD = 0.59).

### 3.1. Secondary School Students

Table 1 summarizes bivariate analyses examining differences among lifetime users and nonusers and, among lifetime users, past 30-day cigarette smokers versus nonsmokers across both age groups. In terms of lifetime cigarette use, correlates included older age (*P* = .02), being male (*P* < .001), past 30-day alcohol use (*P* < .001), lifetime marijuana use (*P* < .001), lower perceived risk of smoking (*P* < .001), less often reading fiction (*P* < .001), more often engaging in sports/exercising (*P* = .02), more often going out in the evening (*P* < .001), less often using the Internet for education or work (*P* < .001), and more often spending time with neighbors and friends (*P* < .001). In the multivariate regression model (Table 2), significant predictors of lifetime cigarette use included being male (*P* < .001), consuming alcohol (*P* < .001), lifetime marijuana use (*P* < .001), and lower perceived risk (*P* = .001).

In terms of past 30-day smoking among lifetime cigarette users (Table 1), correlates included older age (*P* = .04), being male (*P* < .001), past 30-day alcohol use (*P* < .001), lifetime marijuana use (*P* < .001), lower perceived risk of smoking (*P* < .001), less often reading fiction (*P* = .001), less often engaging in sports/exercising (*P* = .04), more often going out in the evening (*P* = .008), less often using the Internet for education or work (*P* = .005), and less often spending time with neighbors and friends (*P* = .01). In the multivariate regression model (Table 2), significant predictors of past 30-day cigarette smoking among lifetime cigarette users included being male (*P* = .03), consuming alcohol (*P* = .05), lifetime marijuana use (*P* = .003), lower perceived risk (*P* < .001), less frequently engaging in sports/exercising (*P* = .009), and more often going out in the evenings (*P* = .05). We also explored interaction effects, specifically gender by activity involvement, and found no significant interactions.

### 3.2. Postsecondary School Students

In terms of lifetime cigarette use among postsecondary school students (Table 1), correlates included being male (*P* < .001), being employed at least part-time (*P* < .001), past 30-day alcohol use (*P* < .001), lifetime marijuana use (*P* < .001), lower perceived risk of smoking (*P* < .001), more often engaging in sports/exercising (*P* = .003), and more often going out in the evening (*P* < .001). In the multivariate regression model (Table 3), significant predictors of lifetime cigarette use included being male (*P* = .001), consuming alcohol (*P* < .001), lifetime marijuana use (*P* < .001), lower perceived risk (*P* < .001), more often going out in the evening (*P* = .003), and using the Internet to listen to music, play, or chat (*P* = .02).

In terms of past 30-day smoking among lifetime cigarette users (Table 1), correlates included being male (*P* < .001), past 30-day alcohol use (*P* = .001), lifetime marijuana use (*P* < .001), lower perceived risk of smoking (*P* < .001), more often going out in the evening (*P* < .001), less often using the Internet for education or work (*P* = .03), and more often spending time with neighbors and friends (*P* = .04). In the multivariate regression model (Table 3), significant predictors of past 30-day cigarette smoking among lifetime cigarette users included being male (*P* = .04), consuming alcohol (*P* = .02), lifetime marijuana use (*P* = .02), lower perceived risk (*P* < .001), and more often going out in the evenings (*P* = .005). We also explored interaction effects, specifically gender by activity involvement, and found no significant interactions.

## 4. Discussion

The current study documented sociodemographic factors and involvement in differing activities as they relate to smoking initiation and progression among secondary and postsecondary students in Tbilisi, Georgia. Key results indicated that this data reflects previously documented findings regarding sociodemographic correlates of smoking, the connection between perceived risk of smoking and smoking initiation and maintenance, and other substance use in relation to smoking. The more novel findings involved leisure time activity involvement and their relation to cigarette smoking.

As found in prior research [8, 9, 12], males were more likely than females to have smoked cigarettes in their lifetime or in the past 30 days. In addition, among those who had smoked cigarettes in their lifetime, males were more likely to be current smokers. Additionally, postsecondary school students were more likely than secondary school students to have smoked in their lifetime or in the past 30 days. Interestingly, however, within these different age groups, age itself was not correlated with lifetime cigarette use or current smoking, which implies that the contextual factors of these settings may have more of an impact on cigarette smoking than age itself. For example, the postsecondary school settings might involve greater freedom of choice, more influence of older peers, changes in social norms around tobacco and other substance use, greater exposure to tobacco marketing, and greater stress or other mental health issues related to the transition [43, 44]. Our findings also indicated that employment and marital status had little or no impact on smoking behavior in this sample of young adults, few of whom were employed (17.2%) or married (4.0%). These findings warrant further examination to understand the psychosocial factors that do, in fact, vary across contexts and impact smoking in Georgia in order to inform tobacco control interventions and policies in this setting.

One factor consistently found to be associated with cigarette use was perceived risk of smoking, which is a well-established correlate [43, 45]. Social norms regarding smoking and perceived risk of smoking have previously been found to be associated [43, 45]. Given the high prevalence of cigarette smoking nationwide in Georgia, these findings are not surprising. This might suggest two intervention targets to reduce the likelihood of smoking initiation and maintenance among youth in Georgia—increasing the perceived harm of smoking and denormalizing cigarette smoking in Georgian youth. In reference to this latter point, it is important to correct any inflated perceptions of smoking prevalence in Georgian youth as well as develop messaging strategies to reduce the social acceptability of smoking in this population.

Using alcohol and marijuana was also highly correlated with lifetime cigarette use and current smoking among both age groups. This is in line with well-established research in other countries documenting the connection between other substance use and smoking [35, 46]. Interestingly, the most high-risk activity related to smoking was going out to parties, cafes, bars, or discos in the evening for both subgroups of youth. Specifically, we found that going to parties and bars was associated with greater likelihood of smoking maintenance among secondary students and with greater likelihood of lifetime cigarette use and smoking maintenance among postsecondary school students. This might imply that these contexts are conducive to substance use in general and cigarette use specifically. In addition, advertising tobacco products through bars, clubs, cafes, and other venues such as these is a highly utilized strategy of the tobacco industry [47–49]. A large number of cross-sectional studies have reported associations between exposure to tobacco marketing and attitudes toward smoking, susceptibility to smoking, smoking experimentation, or regular smoking among youth [43, 50–52]. Thus, exposure to tobacco advertising through these settings and others frequently occupied by youth should be assessed.

We also found that using the Internet to listen to music, play, or chat was associated with greater lifetime cigarette use among postsecondary school students. This may indicate social influence on smoking among young adults; alternatively, this type of Internet use may also indicate a potential for online tobacco advertising. Related to this latter point, new media offers the tobacco industry a powerful and efficient channel for promoting cigarettes and other tobacco products that has largely gone unregulated to date [53]. Evidence of tobacco promotion through online media is emerging, with YouTube, Facebook, and Twitter being prominent sources for tobacco advertising that also has broad reach to youth globally [53]. Assessments of exposure to online tobacco advertising may help distinguish the sources of influence on tobacco use related to Internet use for pleasure.

Engaging in sports and physical activity was associated with reduced likelihood of continued smoking among secondary school students. This suggests that the consistent engagement in physical activity may reduce the likelihood of using cigarettes frequently or continuously, which has been documented in other countries previously [32, 33]. This is likely due to concerns regarding the capacity to engage in these activities if physical functioning is compromised or to social norms among peers being less supportive of smoking [32, 33]. Interestingly, research in other countries has also indicated that athletes often have higher smoking rates [54, 55]. Further examination is needed to understand the role of engaging in physical activity and the potential for greater peer influence in the context of team versus individual sports.

The current findings have important implications for research and practice. In terms of research, this study suggests the need for more research regarding correlates of smoking among youth in Georgia, given the relatively limited scope of factors included in this dataset. Specifically, examining the contextual factors in the two differing school settings (i.e., secondary and postsecondary schools) and the differences among males and females that might contribute to smoking initiation and maintenance are critical. Moreover, the social norms and potential exposure to tobacco marketing should be examined in these differing contexts and how different groups are targeted. Regarding practice, policies involved in the FCTC, particularly those impacting the social norms of smoking (e.g., public smoke-free policies, regulation of tobacco advertising), may influence smoking initiation and maintenance among youth. Moreover, practitioners should understand the relationship between other substance use and smoking behaviors and systematically monitor health behaviors in clinical encounters. Additionally, addressing these behaviors concurrently may prove to be beneficial. Finally, it is important to recognize that smoking prevalence is high among youth in Georgia, and thus, early intervention is critical to address nicotine dependence and smoking-related morbidity and mortality.

### 4.1. Limitations

This study has some limitations. First, this sample was recruited through secondary and postsecondary school students living in Tbilisi, and, thus, we cannot infer how reflective this sample is of the larger youth population in Georgia. Relatedly, we also did not record the school of attendance as a variable. This was done in order to ensure maximum confidentiality of the data given the sensitive nature of the questions (e.g., history of drug use, sexual behavior) and to provide the maximum protection of the schools who participated in the study. It is important to note the large number of school involved (i.e., 24 secondary schools and 13 universities and/or vocational-technical training schools) and thus the greater generalizability of the sample to the larger Tbilisi 15–24-year-old population and potentially the larger population within this age range in Georgia. Despite these strategies, we are uncertain of the extent to which the lifetime and current smoking prevalence accurately reflects actual national or citywide estimates among this population. Second, because of the cross-sectional nature of this study, we cannot determine the directionality of the relationships documented. Furthermore, this was a secondary data analysis of a study examining factors relevant to HIV Prevention. The dataset was not intended to exhaustively assess correlates of tobacco use. Therefore, several important factors potentially related to tobacco use were not assessed, including parental smoking, exposure to tobacco advertising, exposure to secondhand smoke, peer tobacco use, and several other important factors. In addition, the lack of a significant finding regarding the association between marital status, employment, and tobacco use among the postsecondary student population may be due to the relatively small proportions of participants reporting being married or employed. Relatedly, the lack of significant interactions documented in relation to gender may have been due to the small number of females in the cells of current cigarette smokers in both age groups (36 in the 15–18-year-old female group and 79 in the 18–24-year-old female group). Finally, the way in which we operationalized the alcohol and marijuana variables has limitations. However, we examined alternative operationalizations (e.g., continuous variables), and these alternatives did not yield significantly different findings. Despite these limitations, these findings are novel and important as a basis for future research in this area, particularly given the dearth of published research on youth smoking in Georgia.

## 5. Conclusions

Future research should examine contextual factors in secondary and postsecondary schools that impact smoking among Georgian youth. Specifically, factors impacting differential rates of smoking among males and females, the social norms of smoking and other substance use, and the impact of leisure time activity involvement on smoking initiation and maintenance should be examined further. In addition, interventions and policies that might impact attitudes toward smoking and social norms regarding smoking should be investigated and considered.

## Figures and Tables

**Table 1:** Participant characteristics and bivariate analyses examining differences between participants who have never smoked versus have smoked cigarettes at some point in their lifetime and between lifetime cigarette users who have smoked versus not smoked in the past 30 days.

Variable	Total *N* = 1,879 100.0%	15–18 year olds in secondary school						18–24 years enrolled in a postsecondary school					
		Lifetime cigarette use			Smoked in past 30 days^a^			Lifetime cigarette use			Smoked in past 30 days^a^		
		No *N* = 501 56.2%	Yes *N* = 390 43.8%	*P*	No *N* = 233 59.7%	Yes *N* = 157 40.3%^b^	*P*	No *N* = 471 49.4%	Yes *N* = 482 50.6%	*P*	No *N* = 212 44.0%	Yes *N* = 270 56.0%^c^	*P*
	M (SD) or *N* (%)	M (SD) or *N* (%)	M (SD) or *N* (%)		M (SD) or *N* (%)	M (SD) or *N* (%)		M (SD) or *N* (%)	M (SD) or *N* (%)		M (SD) or *N* (%)	M (SD) or *N* (%)	
*Sociodemographics *													
Age (SD)	18.41 (2.38)	16.20 (0.80)	16.32 (0.84)	.02	16.25 (0.79)	16.43 (0.89)	.04	20.48 (1.26)	20.49 (1.29)	.91	20.39 (1.30)	20.56 (1.28)	.16
Gender (%)				<.001			<.001			<.001			<.001
Male	917 (48.8)	182 (36.3)	260 (66.7)		139 (59.7)	121 (77.1)		159 (33.8)	301 (62.4)		110 (51.9)	191 (70.7)	
Female	962 (51.2)	319 (63.7)	130 (33.3)		94 (40.3)	36 (22.9)		312 (66.2)	181 (37.6)		102 (48.1)	79 (29.3)	
Employment (%)^d^		—	—	—	—	—	—			<.001			.20
Unemployed	797 (82.8)							410 (87.0)	379 (78.6)		171 (80.7)	208 (77.0)	
Employed at least part-time	165 (17.2)							61 (13.0)	103 (21.4)		41 (19.3)	62 (23.0)	
Marital status (%)^d^		—	—	—	—	—	—			.46			.06
Not married	924 (96.0)							453 (96.2)	462 (95.9)		207 (97.6)	255 (94.4)	
Married	38 (4.0)							18 (3.8)	20 (4.1)		5 (2.4)	15 (5.6)	
*Other substance use *													
Alcohol use, past month (%)				<.001			<.001			<.001			.001
No	350 (36.4)	237 (47.3)	64 (16.4)		51 (21.9)	13 (8.3)		242 (51.4)	106 (22.0)		61 (28.8)	45 (16.7)	
Yes	612 (63.6)	264 (52.7)	326 (83.6)		182 (78.1)	144 (91.7)		229 (48.6)	376 (78.0)		151 (71.2)	225 (83.3)	
Marijuana use, lifetime (%)				<.001			<.001			<.001			<.001
No	777 (85.5)	466 (98.9)	289 (83.5)		195 (91.5)	94 (70.7)		448 (96.6)	323 (73.6)		171 (84.2)	152 (64.4)	
Yes	132 (14.5)	5 (1.1)	57 (16.5)		18 (8.5)	39 (29.3)		16 (3.4)	116 (26.4)		32 (15.8)	84 (35.6)	
*Psychosocial factors *													
Perceived risk (SD)	12.84 (2.47)	13.62 (2.05)	12.68 (2.45)	<.001	13.23 (2.13)	11.80 (2.68)	<.001	13.46 (2.19)	12.25 (2.57)	<.001	13.08 (2.16)	11.56 (2.69)	<.001
Activity involvement (SD)													
Read fiction literature for entertainment	2.92 (1.17)	3.19 (1.27)	2.82 (1.23)	<.001	2.95 (1.21)	2.62 (1.24)	.009	2.99 (1.15)	2.86 (1.18)	.08	2.91 (1.11)	2.82 (1.23)	.40
Engage in sports, physical activity	3.00 (1.41)	3.31 (1.54)	3.55 (1.46)	.02	3.68 (1.39)	3.37 (1.55)	.04	2.86 (1.47)	3.13 (1.34)	.003	3.01 (1.40)	3.23 (1.29)	.08
Go to parties, cafe, bar or disco in the evening	2.68 (1.11)	2.16 (1.05)	2.53 (1.12)	<.001	2.41 (1.07)	2.72 (1.17)	.008	2.43 (1.07)	2.94 (1.10)	<.001	2.67 (1.06)	3.15 (1.08)	<.001
Use the internet to listen to music, play, chat	4.34 (1.17)	4.60 (0.97)	4.58 (1.00)	.79	4.62 (0.95)	4.53 (1.08)	.41	4.24 (1.24)	4.45 (1.08)	.004	4.47 (1.04)	4.44 (1.11)	.79
Use the internet for educational or work purposes	4.12 (1.19)	3.73 (1.26)	3.32 (1.36)	<.001	3.48 (1.24)	3.09 (1.49)	.005	4.17 (1.13)	4.08 (1.22)	.21	4.21 (1.12)	3.97 (1.29)	.03
Pass time w/neighborhood friends/neighbors	3.04 (1.43)	3.43 (1.40)	3.96 (1.34)	<.001	3.82 (1.37)	3.09 (1.49)	.01	2.96 (1.39)	3.12 (1.47)	.09	2.97 (1.42)	3.24 (1.50)	.04

^a^Among lifetime users. ^b^17.6% of total secondary school participants. ^c^28.3% of total post-secondary school participants. ^d^Among post-secondary school participants.

**Table 2:** Multivariate models examining predictors of lifetime use of cigarettes among participants aged 15–18 years in secondary school and current (past 30 days) smoking among lifetime cigarette users.

Variable	Lifetime cigarette use			Smoked in past 30 days*		
	OR	CI	*P* value	OR	CI	*P* value
*Sociodemographics *						
Age	1.18	0.96, 1.45	.11	1.17	0.85, 1.62	.33
Gender			<.001			.03
Male	Ref	—		Ref	—	
Female	0.38	0.27, 0.53		0.51	0.28, 0.93	
*Other substance use *						
Consumed alcohol, past 30 days			<.001			.05
No	Ref	—		Ref	—	
Yes	3.95	2.69, 5.80		2.29	1.01, 5.24	
Marijuana use, lifetime			<.001			.003
No	Ref	—		Ref	—	
Yes	5.82	2.20, 15.43		3.19	1.50, 6.82	
*Psychosocial factors *						
Perceived risk of smoking	0.88	0.81, 0.95	.001	0.81	0.72, 0.91	<.001
Activity involvement:						
Engage in sports, physical activity	—	—	—	0.77	0.63, 0.94	.009
Go to parties, cafe, bar, or disco in the evening	—	—	—	1.28	1.00, 1.65	.05

*Among lifetime cigarette users.

**Table 3:** Multivariate models examining predictors of lifetime use of cigarettes among participants aged 18–24 years enrolled in a postsecondary school and current (past 30 days) smoking among lifetime cigarette users.

Variable	Lifetime cigarette use			Smoked in past 30 days*		
	OR	CI	*P* value	OR	CI	*P* value
*Sociodemographics *						
Age	1.04	0.90, 1.18	.62	1.08	0.90, 1.30	.40
Gender			.001			.04
Male	Ref	—		Ref	—	
Female	0.54	0.38, 0.77		0.62	0.38, 0.99	
Employment			.08			.35
Unemployed	Ref	—		Ref	—	
Employed at least part-time	1.47	0.95, 2.30		1.31	0.75, 2.29	
Marital status			.64			.11
Not married	Ref	—		Ref	—	
Married	0.83	0.37, 1.84		2.77	0.78, 9.77	
*Other substance use *						
Consumed alcohol, past 30 days			<.001			.02
No	Ref	—		Ref	—	
Yes	2.74	1.92, 3.90		2.09	1.15, 3.77	
Marijuana use, lifetime			<.001			.02
No	Ref	—		Ref	—	
Yes	4.43	2.34, 8.42		1.99	1.13, 3.49	
*Psychosocial factors *						
Perceived risk of smoking	0.82	0.76, 0.89	<.001	0.78	0.71, 0.87	<.001
Activity involvement:						
Go to parties, cafe, bar, or disco in the evening	1.26	1.08, 1.48	.003	1.37	1.10, 1.71	.005
Use the Internet to listen to music, play, or chat	1.22	1.04, 1.42	.02	—	—	—

*Among lifetime cigarette users.
